# Mapping antigenic variation in HIV-1 envelope

**DOI:** 10.1186/1742-4690-10-S1-P32

**Published:** 2013-09-19

**Authors:** Simon Frost

**Affiliations:** 1 University of Cambridge, Cambridge, UK

## 

Recombinant-virus based assays of neutralisation of HIV-1 envelope are routinely used to measure the sensitivity of viruses to neutralisation, and the ability of sera to neutralise viral isolates. However, it is difficult to visualise antigenic differences, especially when the number of viruses and sera are large. Borrowing from developments in antigenic cartography in influenza, I present a statistical model to map viruses and sera into antigenic space, and apply it to data from five published studies of HIV-1 neutralisation. After controlling for potential dose effects, I show that differences in neutralisation of HIV-1 can be mapped into 1-2 dimensions, as ‘broadly-reactive’ sera typically used in neutralisation studies exhibit similar patterns of neutralisation across viral isolates. Antigenic distances generated from the map were associated with amino acid substitutions across many sites scattered over the whole envelope region, suggesting that antigenic differences between viruses arise due to multiple mutations of relatively small effect.

